# Supplementary material for: MOC1 cleaves Holliday junctions through a cooperative nick and counter-nick mechanism mediated by metal ions
Source: Nat Commun. 2024 Jun 17;15:5140. doi: 10.1038/s41467-024-49490-9 (PMC11183143; doi:10.1038/s41467-024-49490-9)
Supplement: Supplementary file 1 — Supplementary Information [file 41467_2024_49490_MOESM1_ESM.pdf]

Supplementary Information

MOC1 cleaves Holliday Junctions through a cooperative nick and counter-nick mechanism mediated by metal ions

D.P. Zhang et al.

Table of Contents

Supplementary Figure 1. Native PAGE analysis of HJ cleavage by MOC1............................ 2

Supplementary Figure 2. The metal-ion triggered catalysis in crystals………………………..3

Supplementary Figure 3. The critical role of K229 in HJ cleavage by MOC1……………...…4

Supplementary Figure 4. Comparison of RuvC active sites from different species……………5

Supplementary Figure 5. The omit map of metal ions bound in the nicked site of the MOC1/nHJ complexes……………………………………………………………………………………….6

Supplementary Figure 6. Gel-based assessment of the cleavage efficiency of MOC1 towards various HJ substrates…………………………………………………………………………7

Supplementary Table 1. Oligos used in this study……………………………………………...…9

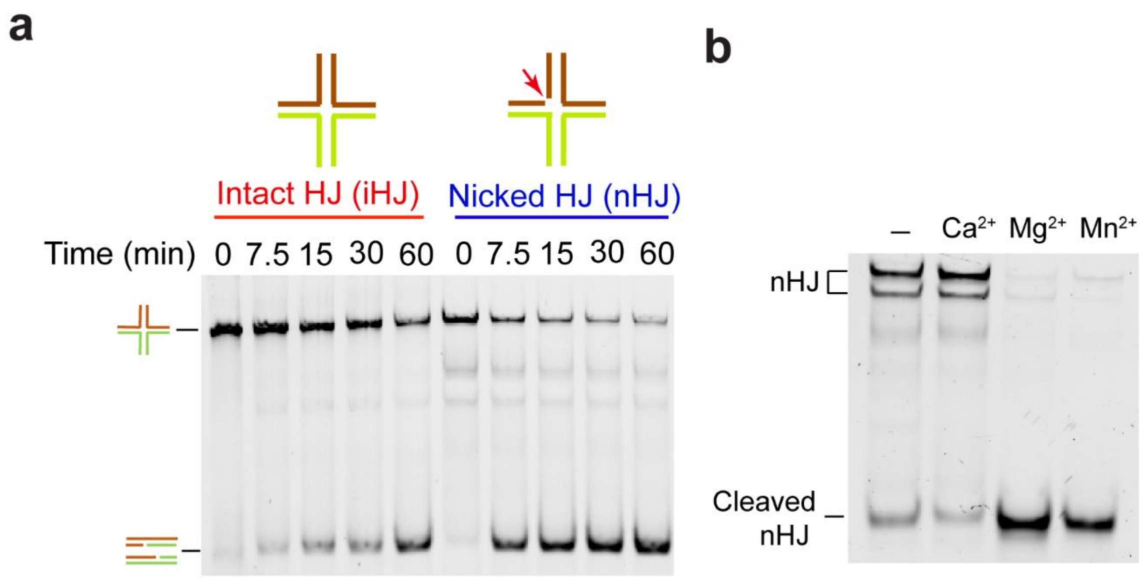

Supplementary Fig. 1 | Native PAGE analysis of HJ cleavage by MOC1. a, A gel-based analysis measuring the cleavage of iHJ and nHJ by MOC1. The reactions were carried out at 30˚C using 175 nM MOC1 and 2 mM Mn^{2+} for indicated time intervals. **b,** The influence of various metal ions on the ability of MOC1 to cleave nHJ. These reactions utilized 175 nM MOC1 and 2 mM Mn^{2+}, conducted at 30˚C for 1 hour. The experiment was independently repeated for three times with similar results. Source data are provided as a Source Data file.

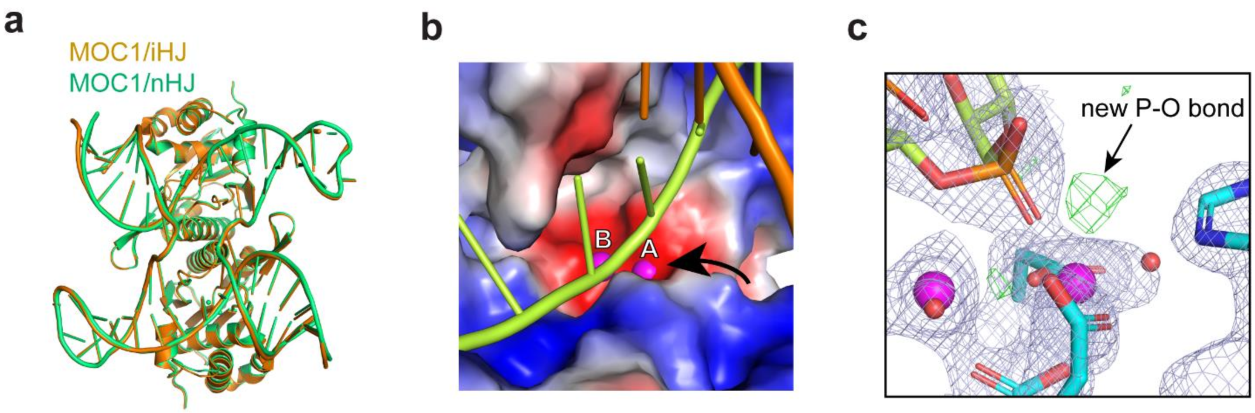

Supplementary Fig. 2│The metal-ion triggered catalysis in crystals. a, A structural comparison between MOC1 complexes with iHJ (orange) (PDB 6IS8)[16] and nHJ (green) (PDB 8KFR from this study). MOC1 is depicted in a cartoon representation and the HJ DNA is illustrated in a ladder model. **b**, Structure of MOC1/nHJ complex bound to two Mn^{2+} ions. HJ is depicted in ladder representation. MOC1 is shown in surface representation, with red, blue and white representing negative, positive and neutral charges, respectively. Mn^{2+} ions are shown in magenta spheres. Arrow indicates the solvent access. **c**, Evidence of product formation following soaking the MOC1/nHJ crystals in Mn^{2+} ions for 180 s. The structure is superimposed with the $2F_o$-F_c electron density map shown in light blue (1.5 σ), and F_o-F_c electron density map shown in green (3.0 σ), respectively.

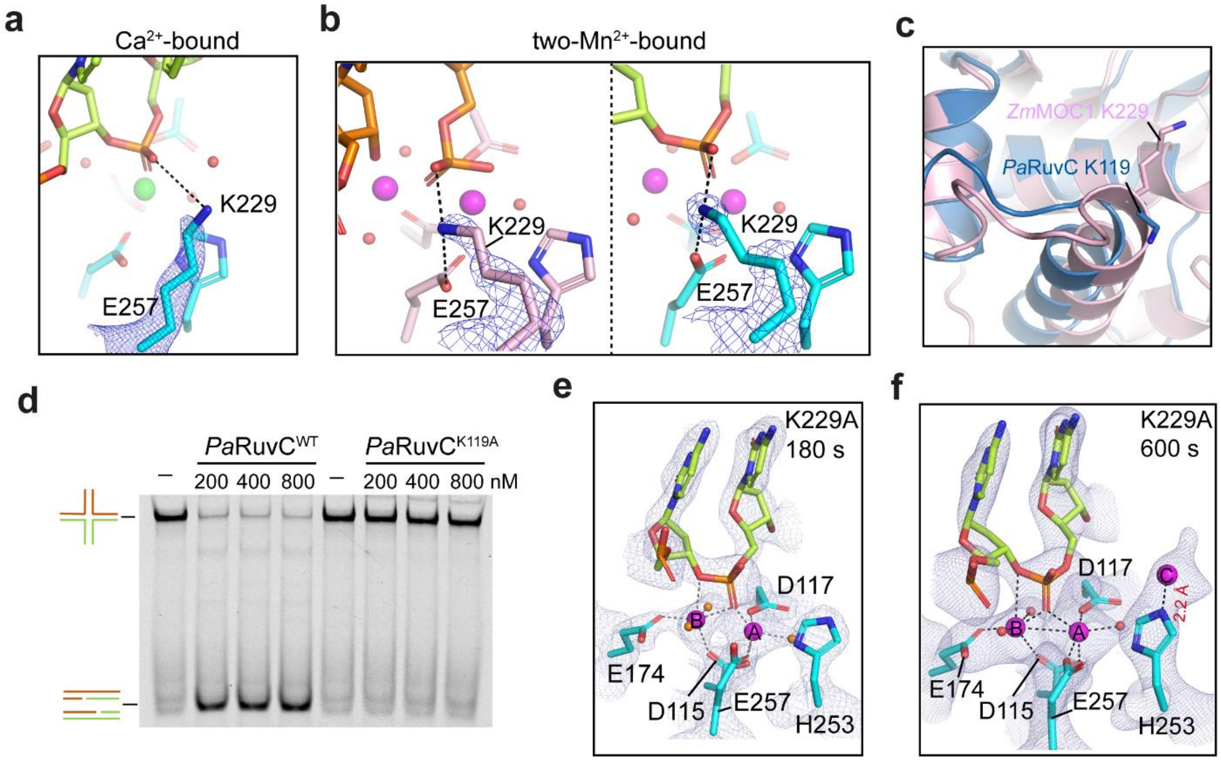

Supplementary Fig. 3 | The critical role of K229 in HJ cleavage by MOC1. a, b, The configurations of K229 in Ca^{2+}-bound (a) (PDB 8KFR from this study) and two Mn^{2+}-bound (b) (PDB 8KFU from this study) states. K229 is superimposed with the $2F_o$-F_c electron density map contoured at 1.0 σ. Hydrogen bonds are indicated by dashed lines. **c**, Structural superposition showing the conserved role of *Zm*MOC1 K229 (pink) and *Pa*RuvC K119 (blue) (PDB 6LW3)[44]. **d**, Effect of K119A mutation on the HJ cleavage by *Pa*RuvC. The reactions were carried out with 2 mM Mn^{2+} and indicated concentrations of *Pa*RuvC at 30˚C for 1 h. The experiment was independently repeated for three times with similar results. Source data are provided as a Source Data file. **e, f**, The configurations of the non-nick site in the $MOC1^{K229A}$/nHJ complex following soaking in Mn^{2+} ions for 180 s (e) (PDB 8KFV from this study) and 600 s (f) (PDB 8KFW from this study). The Mn^{2+} ions depicted in magenta spheres are superimposed with 2*Fo-Fc* electron density map contoured at 1.5 σ. Water molecules are illustrated as orange spheres. The coordination of metal ions and the formation of hydrogen bonds are highlighted with dashed lines.

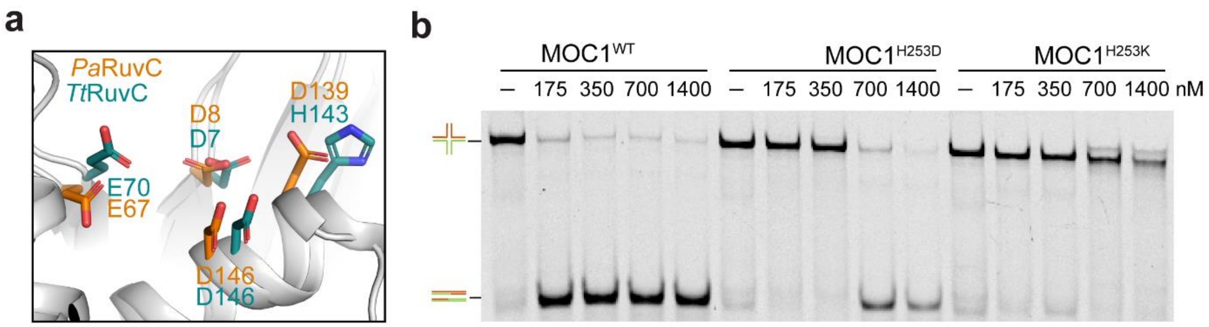

Supplementary Fig. 4│Comparison of RuvC active sites from different species. a, Superposition of the active site of *Pa*RuvC (orange) (PDB 6LW3)[44] and *Thermus thermophilus* (Tt) RuvC (teal) (PDB 4EP4)[33]. Catalytic residues are shown in sticks. **b,** Gel-based analysis of HJ cleavage by WT MOC1 and its variants. The reactions were carried out by incubating 250 nM FAM-labeled HJ DNA, 2 mM Mn^{2+} and indicated concentrations of MOC1 at 30˚C for 1 h. The experiment was independently repeated for three times with similar results. Source data are provided as a Source Data file.

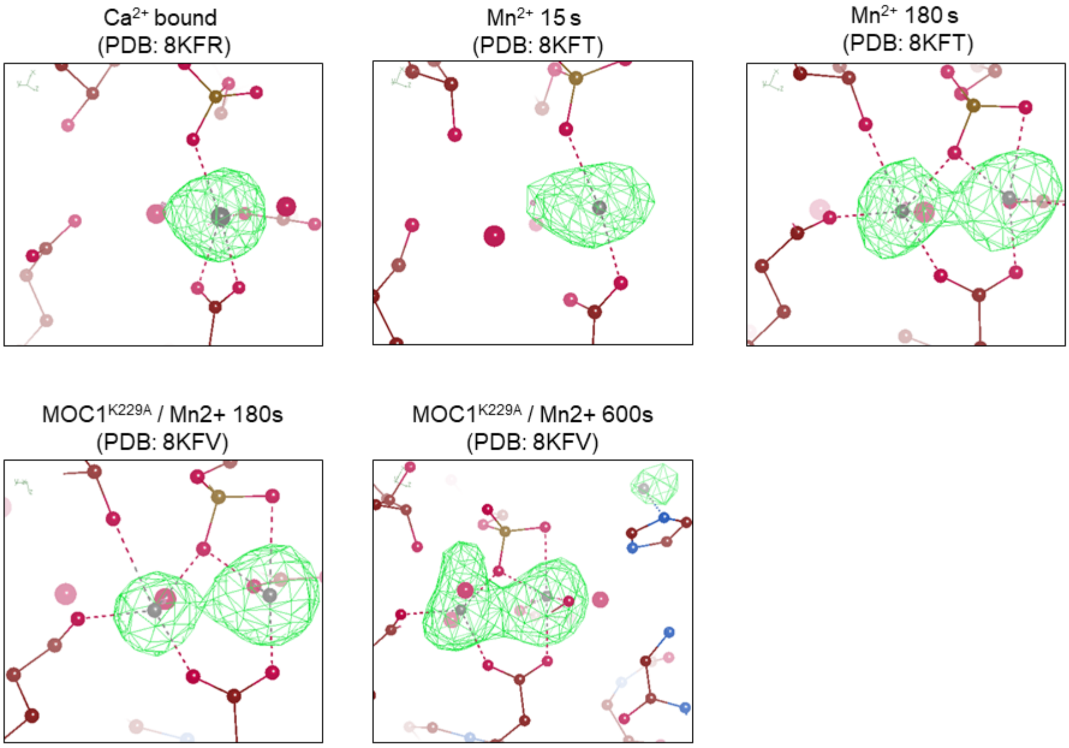

Supplementary Fig. 5 | The omit map of metal ions bound in the nicked site of the MOC1/nHJ complexes. The omit map was generated using Coot and contoured at 5.0 σ.

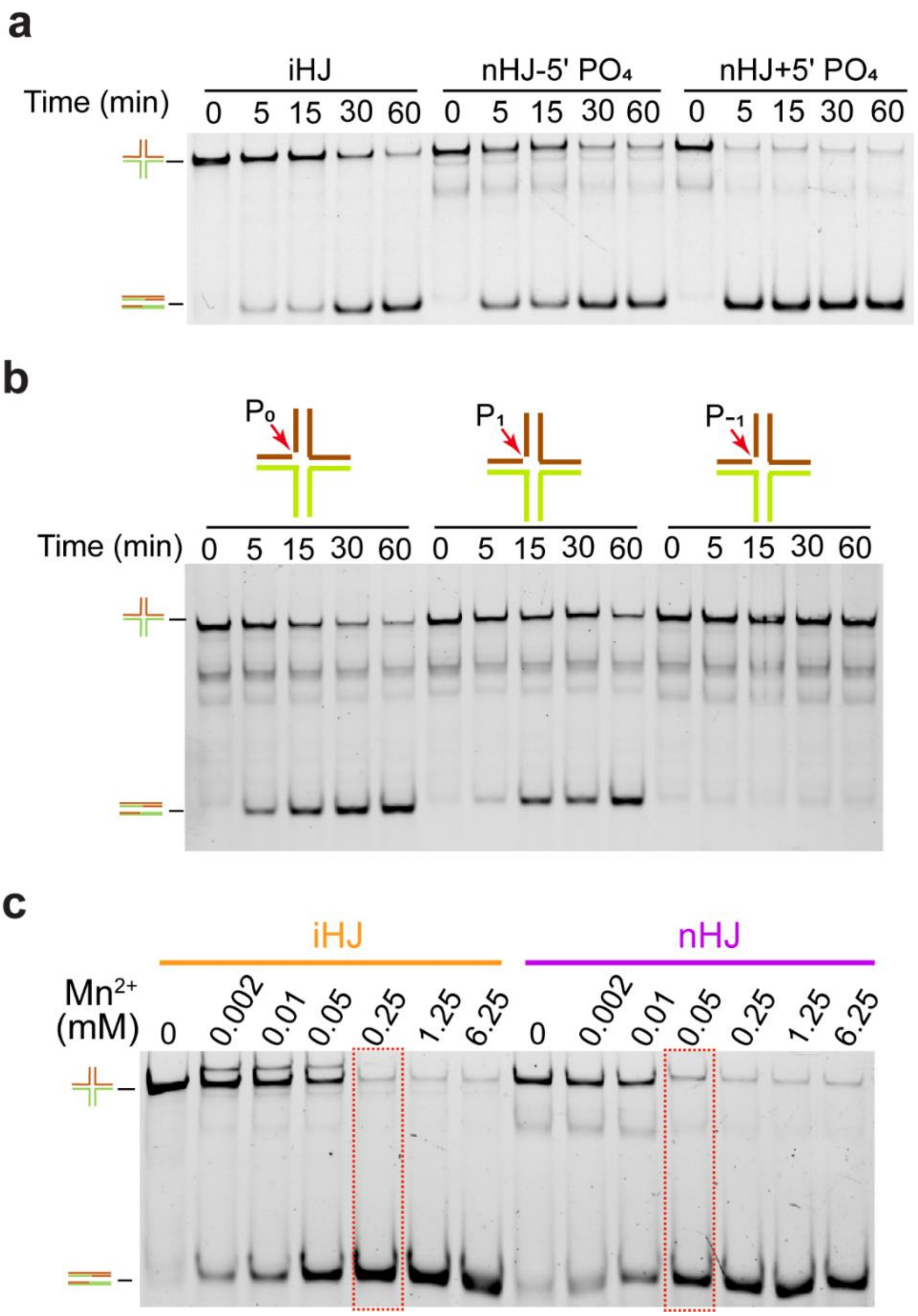

Supplementary Fig. 6 | Gel-based assessment of the cleavage efficiency of MOC1 towards various HJ substrates. a, Effect of the 5′ phosphate absence at the nick site on the cleavage of the second strand. The terms nHJ+5′ PO_4 and nHJ-5′ PO_4 refer to nHJ with and without the 5′ phosphate. **b**, Effects of aberrant cleavage at the first strand

on the cleavage of the second strand. The terms nick@P_0, nick@P_1, nick@P_{-1} denote nHJ DNAs that harbors nicks at positions P_0, P_1 and P_{-1}, respectively. The quantified results from three independent experiments were shown in Fig. 5b. **c**, Mn^{2+}-dose responsive activation of the HJ cleavage by MOC1. These reactions were performed by incubating 175 nM MOC1, 2 mM Mn^{2+} and 250 nM HJ DNA substrates, at 30 ^{o}C for 1 h. The experiment was independently repeated for three times with similar results. Source data are provided as a Source Data file.

Supplementary Table. 1 │ Oligos used in this study.

Oligos used for crystallization	
Oligo-a	5′-CAATCGTGGGAGACCTTTGGTCTCCCTGCAGAT-3′
Oligo-b	5′-ATCTGCAGGGTCTGGTTTCCAGACC-3′
Oligo-c	5′-CACGATTG-3′
Oligos used for FRET-based crystallization	
iHJ-1	5´-ATCTGCAGGGTCTGGTTTCCAGACCCACGATTGTTTCAATCGTAG-GAGACC-3´
iHJ-2	Cy3-5´-GGTCTCCCTGCAGAT-3´-BHQ2
nHJ-1	5´-ATCTGCAGGGTCTGGTTTCCAGACC-3´
nHJ-2	5´- CACGATTGTTTCAATCGTAGGAGACC-3´
Synthetic oligos used for gel-based HJ cleavage assay	
HJ-1	5′-TTGGACGTCCACCCGTATCTGCAGGGTCTGGCCGTGACCATCTTAAGCCG-3′
HJ-2	FAM-5′-CAGCCTAGGAGATCTGCAATCGTGGGAGACCACATTAGCAGTTCGAAATA-3′
HJ-3	5′-CGGCTTAAGATGGTCACGGCCAGACCCACGATTGCAGATCTCCTAGGCTG-3′
HJ-4	5′-TATTTCGAACTGCTAATGTGGTCTCC*CTGCAGATACGGGTGGACGTCCAA-3′

*Denotes the nick site in the nHJ DNA.
