# Supplementary material for: MOC1 cleaves Holliday junctions through a cooperative nick and counter-nick mechanism mediated by metal ions
Source: Nat Commun. 2024 Jun 17;15:5140. doi: 10.1038/s41467-024-49490-9 (PMC11183143; doi:10.1038/s41467-024-49490-9)
Supplement: Supplementary file 3 — Description of Additional Supplementary files [file 41467_2024_49490_MOESM3_ESM.pdf]

Description of Additional Supplementary file

Supplementary Data 1 : Supplementary Data 1 contains the structural and refinement statistics.
